# Understanding the patient experience and treatment benefits in patients with non–small‐cell lung cancer with brain metastasis

**DOI:** 10.1002/cam4.5975

**Published:** 2023-06-12

**Authors:** David Cella, Patrick Y. Wen, Claire Ervin, Susan Vallow, Isabelle Gilloteau, Carla DeMuro, Margaret Mordin, Andrea Chassot Agostinho, Jennifer Dine

**Affiliations:** ^1^ Feinberg School of Medicine Northwestern University Chicago Illinois USA; ^2^ Division of Neuro‐Oncology Dana‐Farber Cancer Institute Boston Massachusetts USA; ^3^ RTI Health Solutions Research Triangle Park North Carolina USA; ^4^ Novartis Services Inc. East Hanover New Jersey USA

**Keywords:** brain metastases, non–small‐cell lung cancer, patient‐reported outcome measures, qualitative interviews

## Abstract

**Background:**

Despite the high prevalence of brain metastases (BM) secondary to non–small‐cell lung cancer (NSCLC) (NSCLC/BM), patients' experiences (symptoms and impacts) are not fully understood. This study sought to understand the patient experience with NSCLC/BM and identify a patient‐reported outcome (PRO) measure fit to capture the most important NSCLC/BM symptoms and impacts.

**Methods:**

A targeted literature review was completed; the National Comprehensive Cancer Network (NCCN)/Functional Assessment of Cancer Therapy–Brain Symptom Index, 24‐item version (NFBrSI‐24) was identified as a relevant measure that assessed the core symptoms and impacts associated with NSCLC/BM. Qualitative interviews composed of concept elicitation and cognitive debriefing with oncologists (*n* = 3) and adult patients (*n* = 16) with NSCLC/BM were conducted to confirm the content validity and evaluate the relevance and appropriateness of the NFBrSI‐24 for this condition.

**Results:**

The NSCLC/BM symptoms and impacts identified in the literature and reported by oncologists and patients were consistent and captured in the NFBrSI‐24. Study participants reported significant burden associated with the symptoms (commonly fatigue, headache) and impacts of NSCLC/BM. Participants indicated that the NFBrSI‐24 captured their most salient experiences with NSCLC/BM and that symptom improvement or a delay in progression, as measured by the NFBrSI‐24, would be meaningful. During the cognitive debriefing, participants generally indicated that the NFBrSI‐24 was comprehensive and easy to understand/answer and that it assessed symptoms they considered most important to treat.

**Conclusions:**

These results suggest that the NFBrSI‐24 adequately captures an appropriate measure of NSCLC/BM symptoms and impact.

## Graphical abstract

The National Comprehensive Cancer Network (NCCN)/Functional Assessment of Cancer Therapy‐Brain Symptom Index, 24‐item version (NFBrSI‐24) was identified as a measure that assessed the core symptoms and impacts associated with NSCLC/BM. Interviews with clinicians and patients found that the NFBrSI‐24 captures the key symptoms and impacts of NSCLC/BM and can be used to assess disease improvements.

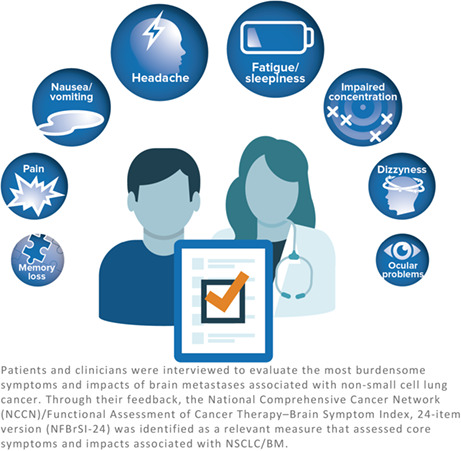

## 1 INTRODUCTION

Lung cancer is the most frequently diagnosed cancer and the leading cause of cancer‐related deaths worldwide; non–small‐cell lung cancer (NSCLC) accounts for approximately 90% of lung cancer cases.^1^, ^2^, ^3^ New therapeutics like immune checkpoint inhibitors are pushing 5‐year survival rates for some patient populations above 20%, but prognosis remains poor for many, and the 5‐year survival rate for patients who are diagnosed in advanced stages hovers between 5%–15%.^3^, ^4^, ^5^ Up to 10% of adults with NSCLC have brain metastases (BM) at initial diagnosis, and 20%–40% of patients with NSCLC develop BM over time.^6^ After development of BM, the median survival drops to 8–10 months, even with active treatment.^4^, ^7^ Beyond the negative impact on patients' survival, there is also a significant and rapid decline in health‐related quality of life (HRQoL) associated with the development of BM.^8^, ^9^

Historically, oncology clinical trial endpoints centered on survival benefits. While prolonging survival is paramount in most situations, it is not the only outcome that patients value.^10^, ^11^, ^12^, ^13^, ^14^, ^15^, ^16^, ^17^ As patients live with terminal cancer longer, there remains a need to maintain their quality of life for as long as possible.^18^ The United States (US) Food and Drug Administration (FDA) and professional organizations such as the International Society of Pharmacoeconomics and Outcomes Research have reported the need to go beyond the traditional oncology endpoints and have encouraged the inclusion of the patient voice in oncology clinical research.^19^, ^20^, ^21^, ^22^, ^23^, ^24^

Despite the prevalence of this condition, and the recent regulatory push for incorporating the patient's voice in clinical research, there is little to no qualitative research providing insight on the patient experience with NSCLC with BM, which can differ from that of patients with NSCLC alone. In addition, it is difficult to accurately assess the full impact of BM on the HRQoL of patients with NSCLC because patients with NSCLC/BM are commonly excluded from oncology clinical trials,^25^ and there is no patient‐reported outcomes measure (PROM) specifically designed for this condition. The FDA requires that all clinical outcome assessments that may be used to support product approval or labeling must be rigorously developed and that the evidence supporting the assessment must include support of content validity in the targeted context of use.^23^ Therefore, a targeted literature review was conducted to identify the most prevalent signs, symptoms, and impacts associated with NSCLC/BM and to identify potentially appropriate PROMs for this context of use.

The National Comprehensive Cancer Network (NCCN)/Functional Assessment of Cancer Therapy–Brain Symptom Index, 24‐item version (NFBrSI‐24) was identified during this review as a candidate measure to include in future clinical trials including patients with NSCLC/BM. The NFBrSI‐24^26^ contains 24 items within four subscales: Physical Disease–Related Symptoms (12 items), Emotional Disease–Related Symptoms (5 items), Treatment Side Effects (5 items), and Functional Well‐Being (2 items). The NFBrSI‐24 was originally developed to measure impacts on patients with primary brain tumors. As such, the measure's content validity within this different context of use (patients with NSCLC/BM) has not been thoroughly evaluated. Therefore, we sought to better understand the patient experience with NSCLC/BM and evaluate the content validity and appropriateness of the NFBrSI‐24 in clinical trials of novel therapeutics for patients with NSCLC/BM.

## 2 METHODS

### 2.1 Study design

This qualitative research project was conducted in a stepwise approach consistent with regulatory recommendations.^23^ The first step was to conduct a targeted literature review to identify the symptoms and impacts reported by patients who had been diagnosed with BM and to determine whether any previously developed patient‐reported instrument adequately assessed these concepts. BM secondary to NSCLC was the focus; however, because of the lack of patient‐reported data in this condition, the search included primary brain tumors and BM secondary to any cancer subtype.

The results of the literature review informed the development of study materials to conduct qualitative interviews with oncologists and patients. Oncologists were interviewed to provide clinical observations of the patient experience with NSCLC/BM, to confirm the results of the literature review, and to provide clinical expert input on the relevance of the NFBrSI‐24 as a measure for patients with NSCLC/BM. Next, semistructured interviews were conducted, including both concept elicitation and cognitive debriefing components to confirm the content validity and appropriateness of the NFBrSI‐24.^27^ These interviews allowed direct elicitation of the symptoms and impacts most important to treat from the patient perspective as well as feedback regarding the appropriateness of the NFBrSI‐24 in this population.^28^ The study was reviewed and approved by the RTI Institutional Review Board.

### 2.2 Literature review and clinical interviews

A targeted literature search was performed in the EvidPRO platform for articles on patient‐reported symptoms associated with BM secondary to any primary malignancy, including primary brain cancer, and PRO measures used to assess BM symptoms and impacts. EvidPRO is a platform that uses Artificial Intelligence (AI) to identify relevant patient‐reported symptoms and PRO instruments from within PubMed for a given search string. The following search strategy was implemented within the Evid Science AI platform:

“Brain Neoplasms”[Mesh] OR brain neoplasm*[Title/Abstract] OR brain tumor*[Title/Abstract] OR brain tumour*[Title/Abstract] OR brain cancer*[Title/Abstract] OR brain carcinoma*[Title/Abstract] OR brain malignan*[Title/Abstract] OR brain metastas*[Title/Abstract]

The Evid Science AI system identified the most salient concepts and PRO measures and organized them into themes or categories with input from an author (M.M.). Searches were run on PROMs for patients with brain tumors or BM and for symptoms associated with NSCLC/BM. The search was refined by researchers to emphasize patient‐reported rather than clinician‐reported outcomes. Once the literature was mined for symptoms and measures, three oncologists with expertise in NSCLC/BM were interviewed via telephone using a semistructured interview guide to build upon and potentially confirm the literature review findings. Interviews were conducted using a semistructured interview guide, which included an open‐ended concept elicitation phase and then a review of the NFBrSI‐24. Specifically, during the interviews, the clinical experts were asked to describe their experiences treating patients with NSCLC with BM (e.g., symptoms, impacts, and unmet needs); the relative importance of these symptoms to treat; and how, if at all, they have measured changes in these symptoms/impacts in the past. Finally, the clinicians were asked to briefly review and discuss the appropriateness of the NFBrSI‐24 in the targeted patient population. Each interview lasted approximately 1 h.

### 2.3 Interviews with study participants

A purposeful sampling strategy^29^, ^30^, ^31^ was used to recruit participants who met the study screening criteria. Patients with self‐reported NSCLC/BM were identified and recruited as study participants through a third‐party global qualitative research firm and screened by a trained medical recruiter to determine eligibility (Table 1). Each participant in this study completed an interview for up to 60 min over the telephone with two experienced qualitative interviewers (C.E. and J.D.), one leading discussion and one collecting field notes. Interviews were recorded and transcribed. During concept elicitation, participants responded to a combination of open‐ended and targeted probe questions to identify a comprehensive listing of NSCLC/BM symptoms and impacts and to explore the relative importance of these symptoms/impacts to treat. Participants were then asked to provide feedback on the NFBrSI‐24 specifically, regarding item relevance and the ease with which they understood and could answer the items. To facilitate the eventual interpretation of NFBrSI‐24 scores, as time permitted, participants were also queried on what would constitute meaningful change as measured by the NFBrSI‐24. The interviewers had no relationship with the study participants.

**TABLE 1:** Participant criteria.

Inclusion criteria
Adults (aged 18 years or older) Diagnosed with stage III or IV NSCLC with BM Read and speak English Willing and able to provide verbal consent for and participate in a 1‐h telephone interview^a^ ECOG Performance Status of 0^b^ or 1^c^ Symptomatic^d^
Exclusion criteria
Individuals who have previously been diagnosed with a different type of primary cancer (i.e., potential interview participant has been diagnosed with a type of cancer other than NSCLC and associated metastases) Individuals who are scheduled to receive external beam radiation between the time of screening and the conduct of the interview

Abbreviations: BM, brain metastases; ECOG, Eastern Cooperative Oncology Group; NSCLC, non–small‐cell lung cancer.

^a^ 1‐hour interviews were reduced to 45 minutes to support participant recruitment and to mitigate interview participant fatigue.

^b^ ECOG 0 = fully active, able to carry on all predisease performance without restriction.

^c^ ECOG 1 = restricted in physically strenuous activity but ambulatory and able to carry out work of a light or sedentary nature (e.g., light housework and office work); at least one symptom of BM (e.g., headache, vomiting, seizures, dizziness, problems with memory, problems with vision or blurred vision, difficulty with physical activities such as walking or balance).

^d^ Participant self‐reported at least one of the following symptoms at screening: headache, vomiting, seizures, dizziness, problems with memory, problems with concentration, vision problems or blurred vision, difficulty with physician activities, and/or fatigue/lack of energy.

### 2.4 Analysis

Data analysis was facilitated by qualitative software (ATLAS‐ti 8.0) and performed by the qualitative interviewers (C.E. and J.D.). Participant demographics and clinical data were analyzed descriptively. Qualitative interview responses were analyzed thematically.^29^, ^32^ Specifically, themes and concepts identified during the concept‐elicitation phase of each interview were compared to identify and confirm the NSCLC BM symptoms and impacts of greatest importance to patients. Similarly, analyses of the cognitive debriefing portion of the interviews were performed using field notes and transcripts, and concepts of importance and potential problems with content or comprehension based on patient input were identified in each interview and then compared with the results of other interviews to document the frequency with which patients reported these concepts and issues.^29^

## 3 RESULTS

### 3.1 Literature review and clinician interviews

A targeted literature search of the EvidPRO platform yielded 1500 articles on patient‐reported BM symptoms, which were mined for salient concepts by Evid Science AI. Symptoms and impacts identified in this search were primarily physical, cognitive, or emotional. Four measures that assess impacts of brain tumors were identified: Functional Assessment of Cancer Therapy–Brain, NFBrSI‐24, Brain Symptom and Impact Questionnaire, and European Organisation for Research and Treatment of Cancer Quality of Life Questionnaire–Brain Tumour Module. The concepts captured in each of these measures were compared to the salient concepts identified in the literature search (Table S1). The NFBrSI‐24 assesses a majority of brain tumor–related symptoms and impacts as well as those symptoms and impacts identified by patients and clinicians as the highest priority to treat. The results of the literature review were confirmed via qualitative interviews with clinical experts (i.e., in that the NFBrSI‐24 assessed concepts most salient to this patient population). The NFBrSI‐24 was therefore ultimately selected for further evaluation with patients.^26^

Interviews were then conducted with 3 oncologists with experience treating patients with NSCLC/BM. During concept elicitation, 14 of the 24 concepts included in the NFBrSI‐24 were spontaneously reported by at least one expert. Four symptoms/impacts were identified by at least two experts as being among the most important symptom to treat. These concepts included muscle weakness/loss of coordination, headache, seizure, and speech problems (e.g., slurring, problems with articulation). Each of these symptoms is assessed in the NFBrSI‐24. Upon review of the NFBrSI‐24, all experts reported that each of the 24 items were relevant to this specific patient population, thus supporting the salience of this measure in patients with BM secondary to NSCLC.

### 3.2 Interviews with patient participants

#### 3.2.1 Participant characteristics

A total of 16 interviews were conducted with eligible participants. The mean age of participants was 58.7 years, and the majority of participants were female (*n* = 13/16) (Table 2). The mean time since NSCLC diagnosis was 3.7 years, while the mean time since BM diagnosis was 2.3 years. Six participants had an Eastern Cooperative Oncology Group Performance Status (ECOG PS)^33^ rating of 0, indicating that they were active and able to carry out all predisease activities without restriction. The remaining participants had an ECOG PS of 1, defined as being restricted in physically strenuous activity but able to ambulate and carry out work of a light or sedentary nature (e.g., light housework and office work), and at least one symptom of BM (e.g., headache, vomiting, seizures, dizziness, problems with memory, problems with vision or blurred vision, difficulty with physical activities such as walking or balance).

**TABLE 2:** Participant characteristics.

Characteristic	ECOG = 0 (*n* = 6)	ECOG = 1 (*n* = 10)	Total (*N* = 16)
Age, mean (SD, range), years	55.8 (11.5, 42–73)	60.4 (8.2, 45–68)	58.7 (9.5, 42–73)
Gender, *n* (%)			
Male	1 (16.7)	2 (20.0)	3 (18.8)
Female	5 (83.3)	8 (80.0)	13 (81.3)
Duration since NSCLC diagnosis, mean (SD, range), years	3.5 (2.2, 0.9–6.9)	3.9 (2.8, 0.7–8.4)	3.7 (2.5, 0.7–8.4)
Duration since BM diagnosis, mean (SD, range), years	3.0 (1.4, 0.9–4.8)	1.9 (1.8, 0.3–4.6)	2.3 (1.7, 0.3–4.8)
Current treatment/medications, *n* (%)^a^			
Radiation	5 (83.3)	5 (50.0)	10 (62.5)
Chemotherapy	2 (33.3)	7 (70.0)	9 (56.3)
Opioid	—	6 (60.0)	6 (37.5)
Steroid	2 (33.3)	3 (30.0)	5 (31.3)
Nonchemotherapy anticancer drugs	2 (33.3)	—	2 (12.5)
Surgery	1 (16.7)	—	1 (6.3)
Laser interstitial thermal therapy	1 (16.7)	—	1 (6.3)
Immunotherapy	1 (16.7)	—	1 (6.3)
Race/ethnicity, *n* (%)			
American Indian	—	1 (10.0)	1 (6.3)
Black	1 (16.7)	2 (20.0)	3 (18.8)
White	5 (83.3)	7 (70.0)	12 (75.0)
Able to live independently, *n* (%)			
Yes	6 (100.0)	7 (70.0)	13 (81.3)
No	—	3 (30.0)	3 (18.8)
Current living situation, *n* (%)			
With spouse/partner	6 (100.0)	9 (90.0)	15 (93.8)
Alone	—	1 (10.0)	1 (6.3)
Highest level of education, *n* (%)			
College degree	2 (33.3)	4 (40.0)	6 (37.5)
Some college	2 (33.3)	3 (30.0)	5 (31.3)
Graduate degree	1 (16.7)	2 (20.0)	3 (18.8)
Some graduate school	—	1 (10.0)	1 (6.3)
High school	1 (16.7)	—	1 (6.3)
Employment status, *n* (%)^b^			
Unable to work due to disability	2 (33.3)	7 (70.0)	9 (56.3)
Retired	2 (33.3)	4 (40.0)	6 (37.5)
Unemployed	1 (16.7)	1 (10.0)	2 (12.5)

Abbreviations: BM, brain metastases; ECOG, Eastern Cooperative Oncology Group; NSCLC, non–small‐cell lung cancer; SD , standard deviation.

^a^ Self‐reported treatments in the past 3 months.

^b^ One participant reported both “retired” and “unable to work due to disability.”

#### 3.2.2 Concept elicitation

##### Symptoms

**FIGURE 1:**
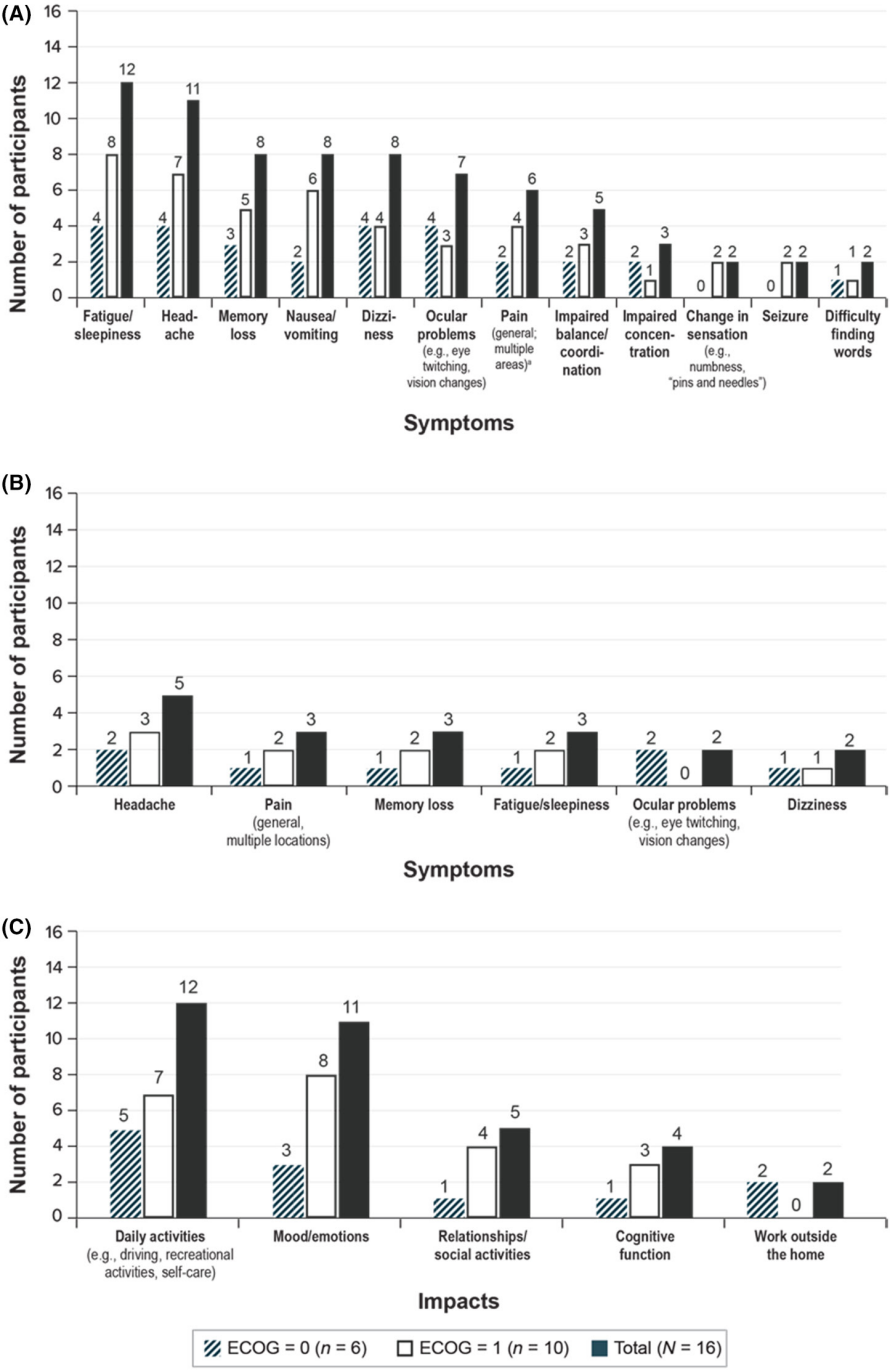
Symptoms and impacts reported by participants. (A) Symptoms spontaneously reported by ≥2 participants. (B) Most bothersome symptoms as reported by participants. (C) Impacts of non–small‐cell lung cancer with brain metastases reported by participants. ECOG, Eastern Cooperative Oncology Group.

The most common spontaneously reported symptoms of BM were fatigue or sleepiness (*n* = 12/16) and headaches (*n* = 11/16); the least reported symptoms were change in sensation, seizures, and difficulty finding words (*n* = 2/16 for each; Figure 1A). The majority of participants (*n* = 12/16) reported having experienced at least some of these symptoms prior to diagnosis with BM.

The symptoms most frequently reported spontaneously by participants were also identified as the most bothersome: headache (*n* = 5/16), pain (*n* = 3/16), memory loss/difficulty (*n* = 3/16), and fatigue (*n* = 3/16; Figure 1B). Each of these symptoms were described as very difficult for patients to alleviate and as having a significant negative impact on their ability to perform daily activities. Headache (*n* = 8/16) and fatigue (*n* = 7/16) were also ranked highest among the symptoms participants reported as most important to treat (Table 3). Representative quotes are found in Table 4.

**TABLE 3:** Top three most important symptoms to treat reported by ≥2 participants (*N* = 16).

Symptom	Single most important,			Among the Top 3,		
	ECOG = 0 (*n* = 6)	ECOG = 1 (*n* = 10)	Total, *N* (%)	ECOG = 0 (*n* = 6)	ECOG = 1 (*n* = 10)	Total, *N* (%)
Headache	1	4	5 (31.0)	3	5	8 (50.0)
Fatigue/sleepiness	0	3	3 (18.8)	2	5	7 (43.8)
Memory loss	0	0	0 (0.0)	1	2	3 (18.8)
Ocular problems (e.g., eye twitching, vision changes)	1	0	1 (6.3)	3	0	3 (18.8)
Pain (general, multiple locations)	0	1	1 (6.3)	0	3	3 (18.8)
Nausea/vomiting	1	1	2 (12.5)	1	2	3 (18.8)
Dizziness	0	1	1 (6.3)	1	2	3 (18.8)
Impaired concentration	2	0	2 (12.5)	2	0	2 (12.5)
Impaired balance/coordination	0	0	0 (0.0)	0	2	2 (12.5)

*Note*: One participant was unable to identify a single most important symptom to treat. All other participants identified at least one most important symptom to treat.

Abbreviation: ECOG, Eastern Cooperative Oncology Group.

**TABLE 4:** Participant quotes describing symptoms and impacts.

Symptoms
*Fatigue/sleepiness*
Some days, I just feel very lethargic. I just don't have the energy. My body is weak The fatigue has been with me for a long time. It takes me forever to go to sleep. And then I'm up in an hour I would lay down, and I would go to sleep. I was just so exhausted Very weak in my arms and my legs. I tend to be sleeping a lot, trying to conserve energy My body just feels heavy. My eyes want to close I spend a lot of time resting on the couch. I'm not active, out doing hobbies and chores. And I'm quite sick I woke up already tired… I do something and then I have to lay down
*Headache*
Headache … if I could probably get rid of this headache, probably [with] the other symptoms, I can function Really bad headaches. It's sometimes really hard for me to think or to focus Reading used to be enjoyable, but it more or less hurts my head, so I try to not be that strenuous on my eyes because then I get a headache
*Memory loss*
It's probably the memory part. I don't like losing control of that and I've always been able to capture things and retain them and I'm not able to do that I can't remember details, and sometimes I forget parts of conversations I've had What's frustrating is being forgetful… What's frustrating is being forgetful or feeling like I'm not part of the conversation due to fading in and out a little bit, so to speak. I don't feel like my old self If the doctor asks a question that I can't remember, then [my husband] usually chimes in It's like [the information is] stuck, and I have to look for it, and it bothers me so much if I forget something
*Nausea/vomiting*
I had several episodes where I would end up in the ER. [I] would be vomiting… I could not even hardly hold my head up Very nauseous, my stomach just always feels very uneasy I had nausea and vomited
*Dizziness*
I was so dizzy I could barely walk. And I actually fell down the stairs It feels like the whole world is spinning around me… And then, I usually get ill [nauseated and vomit] When I get up, I'm a little dizzy. I just go sit down again. And you have a little blackout session when it's real bad
*Ocular problems* (*e.g., eye twitching, vision changes*)
Sometimes I can't see very good So, blurriness, sometimes I'm not able to see
*Pain* (*general; multiple areas*)
I had a lot of backache I had…right shoulder pain
*Impaired balance*
I have a little bit of a balance problem. It's not dizziness, but I'm a little tipsy or something I don't have correct balance. I can't ride a bike…
*Coordination*
Coordination… I was unable to walk unassisted
Impacts
*Social activities*
It's hard to be around any kind of conversation for a long time, even on watching television, which just wears me out. My husband tries to sit and have a conversation with me, and I just can't do it sometimes
*Daily activities*
I used to walk a lot. And it stopped me from doing this because I'm afraid to fall It'd hurt to go to the bathroom because I had a huge head rush… I can't even really describe how painful it was At this point my wife helps out with me doing certain things, bathing I used to walk a lot. And it stopped me from doing this because I'm afraid to fall It'd hurt to go to the bathroom because I had a huge head rush… I can't even really describe how painful it was I can't concentrate on reading. It's been really tough I keep an immaculate home, so I struggle through the fatigue and do what I can
*Mood and emotions*
This will probably be my last holiday. I'm depressed I'm miserable, and no, I don't do anything anymore It makes me feel bad that I missed a lot of things, you know? I'll get a little depressed, saying, ‘Why'd this happen to me?’ People say I'm more irritable
*Social and familial relationships*
I might ask more questions and not remember the answer … people get annoyed with me I can't participate with my family and interact the way I would like to Nobody really wants to be around me anymore… It's hard to be around any kind of conversation for a long time, even on watching television, which just wears me out

Of note, a total of 19 potential symptoms associated with BM secondary to NSCLC were reported across the first eight interviews. No new symptoms were reported across the final eight interviews, thus indicating that concept saturation was achieved with respect to symptoms of BM secondary to NSCLC.

##### Impacts

The most frequently reported impacts associated with BM centered on daily activities (e.g., driving, recreational activities, and self‐care; *n* = 12/16) and decrements in mood or emotions (*n* = 11/16; Figure 1C). Several participants reported struggling with daily tasks and in some cases had become dependent on others for assistance performing certain activities, including driving, walking, and bathing. Representative quotes are presented in Table 4.

The realities of living with advanced‐stage metastatic cancer resulted in many participants struggling emotionally. Participants reported feeling anxiety, sadness, and anger about their condition.

As shown in participant quotes in Table 4, some participants (*n* = 5/16) reported a negative impact on their social and familial relationships because of their BM symptoms.

#### 3.2.3 Treatment targets

Participants were asked how meaningful, if at all, a treatment would be that could delay symptom progression. Three‐quarters of participants (*n* = 12/16) reported that a treatment that would slow or delay symptom progression would be meaningful and valuable, noting any delay would allow them to maintain a better HRQoL, as shown by the following quote:

“Yeah, bring that [treatment] on. I don't care if it helps me for one minute. There's one thing you learn when you're in this situation, every second is precious.”

The remaining participants (*n* = 4/16) reported that delaying symptom progression was not meaningful due to the severity of the impact of their current symptoms. They placed higher priority on treatments for their primary and secondary tumors themselves, resulting in reduction of tumor size or prolonged life expectancy, over symptom improvements:

I don't think it would be [meaningful] for me and my situation. I don't think I have a lot of time, and slowing it down wouldn't improve what time I have left.

#### 3.2.4 Cognitive debriefing

The majority of participants indicated that the NFBrSI‐24 assessed symptoms and impacts relevant to their experiences and that the items themselves were generally clear and easy to understand and answer. Their responses are summarized in Table 5. When asked to provide feedback specifically regarding the instructions and recall period, each participant noted that the instructions were easy to understand and follow as written; however, a few participants noted that a shorter recall period (i.e., shorter than 1 week) would improve response accuracy. Participants also generally reported that the response scale was easy to use, that each of the options was distinct, and that the options covered the full range of their experiences.

**TABLE 5:** Participant quotes regarding the cognitive debriefing of the NFBrSI‐24.

Quotes
**Instructions** [Example probes: “In your own words, what are these instructions asking you to do? Is there anything we could do to make them clearer or easier to understand?”
It's easy. It's easy to read and easy to respond back to They're asking me to qualify what each question is from 0 to 4, not at all to very much. Pretty simple They're pretty easy to understand… Well, it wasn't too hard at all [to understand]
**Symptoms and Experiences** [Example probes: Are there any important symptoms/impacts that are not covered in this questionnaire? If so, which one(s)?
Very thorough. I've gotten a lot of questionnaires at doctors, and these are more direct questions rather than general questions… You pretty much covered everything I'm going through. No [nothing is missing]…, it did a real good job.
Recall period [Example probes: The instructions ask you to think about your experiences over the past 7 days. How easy or difficult is that for you?]
I can remember the last 7 days. It's easy. Seven days, yeah. I mean, it might be a little hard for me sometimes because it can kind of become pretty blurred. A little difficult to think [about the past] 7 days. I actually just [thought] about the last couple of days.
Response options [Example probes: Looking at these answer choices, how easy or difficult is it to select an accurate response? Why? Are there too many or too few choices? Is each response relatively distinct?
It's easy [to select a response] I think it's [the response scale] pretty easy and clear They're [response options] all different… Four [‘very much’], I consider every day all day. Three [‘quite a bit’] is just most of the time. Somewhat is like maybe often but not so often It seems they’ve provided enough, just right
Meaningful improvements [Example probes: If a treatment moved you from *[response option]* to *[adjacent response option]*, how meaningful if at all would that improvement be?]
**If you were taking a treatment, and it moved you from a 4 to a 3, how meaningful, if at all, would that be?** It would mean a lot because some progress is better than none. I think any little bit would help. I think any gain would be good. Anything that you can do to lessen the amount of time I'm having these issues is an improvement, so ‘quite a bit’ is an improvement from ‘very much.’

*Note*: Bold text indicates interviewer probe.

The majority of participants (*n* = 10) indicated that the NFBrSI‐24 was a comprehensive assessment tool of the symptoms and experiences associated with NSCLC/BM.

Although six participants reported at least one symptom as missing, these missing symptoms varied and did not overlap among participants. These potentially missing symptoms included dizziness, vision changes, negative impact on sex life, generalized pain, impact on daily life in general, and short‐term memory.

Thirteen of the 16 participants were probed regarding what would constitute a meaningful change across different NFBrSI‐24 items. Almost all (*n* = 12/16) participants indicated that a 1‐ to 2‐point improvement on the 5‐point scale would indicate a meaningful improvement on the items for which this probe was posed.

## 4 DISCUSSION

This study ultimately supported the content validity of the NFBrSI‐24, a PROM initially developed for patients with primary brain tumors, for use in patients with NSCLC/BM. Specifically, both clinicians and patients described the measure's ability to assess the core symptoms and impacts of BM secondary to NSCLC. In addition, study participants reported that changes as measured by the NFBrSI‐24 would represent meaningful improvements in their lives. To the best of our knowledge, this study is one of the first to examine the patient‐reported experience associated with NSCLC/BM for the identification of an appropriate PROM. Not surprisingly, these patients reported multiple symptoms and impacts, and although increase in survival was paramount, symptomatic interview participants clearly described a desire for symptom improvement or a delay in symptom progression. These data suggest there is a significant unmet need in this area of study and treatment as patients balance their desire for longevity with HRQoL.

Regulatory agencies and patients are interested in treatments that not only improve survival but improve lives—specifically treatments that reduce or delay worsening of symptoms and impacts reported as important to treat. When developing novel therapies, it is important to understand the symptoms and impacts experienced by patients, the relative importance of these symptoms to treat (from the patient perspective), and the outcome that would constitute a meaningful treatment benefit across these symptoms and impacts.

Patient‐reported outcome measures for this context of use are limited. One of the potential reasons for this scarcity of instruments could be that patients with NSCLC/BM are mostly excluded from clinical trials.^25^ Recognizing this unmet need, the FDA issued industry guidance in 2020 encouraging the inclusion of patients with BM in clinical trials.^34^ With this remit and the clear need for effective treatments for this patient population, there also comes the need for a rigorously developed measure to capture patient experiences. The results of this qualitative study suggest that the NFBrSI‐24 is suitable for this purpose. Specifically, based on the review of the literature and qualitative interviews with both patients and clinicians, the NFBrSI‐24 was reported to assess the major symptoms and impacts associated with NSCLC/BM and was generally reported as clear and easy to understand and answer. Similarly, patients who participated in this study reported that the NFBrSI‐24 was a comprehensive assessment of their symptoms regardless of ECOG performance status. These findings further support (1) the content validity of the NFBrSI‐24 for this context of use and (2) this measure's potential fit for the purpose of assessing symptoms and impacts associated with NSCLC/BM in clinical trials. Collectively, the results of this study are in general alignment with the research conducted during the NFBrSI‐24 development,^26^ notably with respect to the identification of symptoms deemed most bothersome and most important to treat by adults with BM. To confirm the quantitative properties of the measure (e.g., construct validity, reliability, and responsiveness) and the interpretation of NFBrSI‐24 scores (including meaningful change) in this context of use, a rigorous psychometric evaluation of the NFBrSI‐24 using clinical trial data is recommended.

Despite the rigorous approach employed in this study, some limitations remain. The sample size was relatively small, and 13 out of 16 patients identified as female. However, concept saturation,^24^, ^35^ defined as the point at which no new important concepts are gleaned from the qualitative interviews, was reached, indicating the sample size was adequate for this purpose. Nevertheless, evaluation of the NFBrSI‐24's performance in a larger sample will be informative. For this study, only adults with ECOG PS scores of 0 or 1 were included, in order to match, as much as possible, planned clinical trials. Patients with more advanced disease, including those with ECOG PS scores of 2–3, may have different symptoms or concerns, particularly regarding the value of a treatment that delays symptom progression. Similarly, a patient's previous treatment regimen experience could potentially influence their ongoing symptoms and impacts as well as their perception of treatment benefit. Although treatment experience was collected at screening, patient experience and treatment expectations were not examined by prior treatment experience.

## 5 CONCLUSION

The results of this qualitative study support the content validity and comprehensibility of the NFBrSI‐24 in patients with BM secondary to NSCLC. The NFBrSI‐24 is appropriate for use in future clinical trials, the results of which will further inform its validity.

## AUTHOR CONTRIBUTIONS

**David Cella:** Formal analysis (equal); writing – review and editing (equal). **Patrick Wen:** Formal analysis (equal); writing – review and editing (equal). **Claire Ervin:** Conceptualization (equal); data curation (equal); formal analysis (equal); funding acquisition (equal); investigation (lead); methodology (equal); supervision (lead); writing – review and editing (equal). **Susan Vallow:** Conceptualization (equal); formal analysis (equal); methodology (equal); writing – review and editing (equal). **Isabelle Gilloteau:** Conceptualization (equal); funding acquisition (equal); writing – review and editing (equal). **Carla DeMuro:** Data curation (equal); investigation (equal); writing – review and editing (equal). **Margaret Mordin:** Data curation (equal); formal analysis (equal); investigation (equal); writing – review and editing (equal). **Andrea Chassot Agostinho:** Formal analysis (equal); writing – review and editing (equal). **Jennifer Dine:** Conceptualization (equal); data curation (equal); formal analysis (equal); funding acquisition (equal); investigation (equal); methodology (equal); project administration (lead); writing – review and editing (equal).

## FUNDING INFORMATION

Novartis Pharmaceutical Inc. provided the financial support for the study. RTI Health Solutions, an independent nonprofit research organization, received funding under a research contract with Novartis to conduct this study and provide publication support in the form of manuscript writing, styling, and submission.

## CONFLICT OF INTEREST STATEMENT

JD, CE, CD, and MM are full‐time employees of RTI Health Solutions, an independent nonprofit research organization, which was retained by Novartis Pharmaceuticals Corporation to conduct the research which is the subject of this manuscript. Their compensation is unconnected to the studies on which they work. SV, IG, and ACA are employees of Novartis Pharmaceutical Corporation and hold shares and/or stock options in the company. DC is President of FACIT.org. PYW has received research support from AstraZeneca/Medimmune, BeiGene, Celgene, Chimerix, Eli Lily, Genentech/Roche, Kazia, MediciNova, Merck, Novartis, Nuvation Bio, Puma, Servier, Vascular Biogenics, and VBI Vaccines; and serves on advisory boards for AstraZeneca, Bayer, Black Diamond, Boehringer Ingelheim, Boston Pharmaceuticals, Celularity, Chimerix, Day One Bio, Genenta, GlaxoSmithKline, Insightec, Karyopharm, Merck, Mundipharma, Novartis, Novocure, Nuvation Bio, Prelude Therapeutics, Sapience, Servier, Sagimet, Vascular Biogenics, and VBI Vaccines.

## Supporting information


Data S1.

## Data Availability

Data are primarily in the form of transcripts and cannot be made available in order to protect participant privacy in accordance with the principles of the Belmont Report.
