# Supplementary material for: Understanding the patient experience and treatment benefits in patients with non–small‐cell lung cancer with brain metastasis
Source: Cancer Med. 2023 Jun 12;12(12):13637–48. doi: 10.1002/cam4.5975 (PMC10315826; doi:10.1002/cam4.5975)
Supplement: Supplementary file 1 — Data S1. [file CAM4-12-13637-s001.docx]

Understanding the Patient Experience and Treatment Benefits in Patients With Non–Small-Cell Lung Cancer With Brain Metastasis

**Running title:** Interviews With NSCLC Patients With BM

David Cella, PhD^1^

Patrick Y. Wen, MD^2^

Claire Ervin, MPH^3^

Susan Vallow^4^

Isabelle Gilloteau^4^

Carla DeMuro^3^

Margaret Mordin, MS^3^

Andrea Chassot Agostinho, MD^4^

Jennifer Dine, PhD, RN^3^

^1^Feinberg School of Medicine, Northwestern University, Chicago, Illinois; ^2^Division of Neuro-Oncology, Dana-Farber Cancer Institute, Boston, MA; ^3^RTI Health Solutions, Research Triangle Park, NC; ^4^Novartis Services Inc., East Hanover, NJ

Corresponding Author:

Claire Ervin

RTI Health Solutions,

3040 East Cornwallis Road,

PO Box 12194

Research Triangle Park, NC 27709

cervin@rti.org

# Supporting Information

Table 1. Mapping of Concepts Across Brain Cancer Specific PRO Measures Identified in the Literature

| Concept |  | NFBrSI-24 | FACT-Br | BASIQ | EORTC  QLQ-BN20 |
| --- | --- | --- | --- | --- | --- |
| Headaches |  | ✓ | ✓ | ✓ | ✓ |
| Trouble with coordination |  | ✓ | ✓ | - | ✓ |
| Had seizures |  | ✓ | ✓ | - | ✓ |
| Need help for self-care |  | ✓ | - | ✓  (bathing, getting dressed) | - |
| Weakness in arms/legs |  | ✓ | ✓ | ✓ (physically weak) | ✓ |
| Losing weight |  | ✓ | - | - | - |
| Trouble meeting needs of family |  | ✓ | - | - | ✓ (concern about) |
| Difficulty expressing thoughts |  | ✓ | ✓ | - | ✓ |
| Sleeping well |  | ✓ | - | - | - |
| Able to concentrate |  | ✓ | ✓ | ✓ | - |
| Able to remember new things |  | ✓ | ✓ | ✓ | - |
| Able to find the right words |  | ✓ | ✓ | ✓ | ✓ |
| Bothered by change in personality |  | ✓ | ✓ | - | - |
| Worry condition will get worse |  | ✓ | - | - | ✓ |
| Afraid of having seizure |  | ✓ | ✓ | - | - |
| Frustrated |  | ✓ | ✓ | - | - |
| Losing hope in fight against illness |  | ✓ | - | - | - |
| Lack of energy |  | ✓ | - | ✓ | ✓ |
| Nausea |  | ✓ | - | ✓ | - |
| Bothered by side effects |  | ✓ | - | - | - |
| Feel fatigued |  | ✓ | - | ✓  (tired) | - |
| Good appetite |  | ✓ | - | - | - |
| Able to enjoy life |  | ✓ | - | - | - |
| Content with quality of life right now |  | ✓ | - | - | - |
| Trouble with eyesight |  | - | ✓ | ✓ | ✓ (double; blurry) |
| Feel independent |  | - | ✓ | - | - |
| Trouble hearing |  | - | ✓ | - | - |
| Able to make decisions and take responsibility |  | - | ✓ | - | - |
| Bothered by drop in contributions to family |  | - | ✓ | - | - |
| Able to put thoughts together |  | - | ✓ | - | - |
| Able to read like used to |  | - | ✓ | ✓ | ✓ |
| Able to write like used to |  | - | ✓ | - | - |
| Able to drive vehicle |  | - | ✓ | - | - |
| Trouble feeling sensations in arms, hands, legs |  | - | ✓ | ✓ (numbness) | - |
| Dizziness |  | - | - | ✓ | - |
| Following a story |  | - | - | ✓ | - |
| Walking |  | - | - | ✓ | - |
| Doing things around the house |  | - | - | ✓ | - |
| Difficulty speaking |  | - | - | - | ✓ |
| Hair loss |  | - | - | - | ✓ |
| Itching of skin |  | - | - | - | ✓ |
| Unsteady on feet |  | - | - | - | ✓ |
| Trouble controlling bladder |  | - | - | - | ✓ |

NFBrSI-24 = NCCN/Functional Assessment of Cancer Therapy-Brain Symptom Index – 24 item version; FACT-Br = Functional Assessment of Cancer Therapy-Brain subscale; BASIQ = Brain Metastases Symptom and Impact Questionnaire; EORTC = European Organization for Research and Treatment of Cancer
